# Effects of AR7 Joint Complex on arthralgia for patients with osteoarthritis: Results of a three-month study in Shanghai, China

**DOI:** 10.1186/1475-2891-7-31

**Published:** 2008-10-27

**Authors:** Qingwen Xie, Rong Shi, Gang Xu, Lifu Cheng, Liyun Shao, Jianyu Rao

**Affiliations:** 1 School of Public Health, Shanghai Jiao Tong University, Shanghai 200025, PR China; 2 Zha Pu Community Health Service Center of Shanghai, Shanghai 200080, PR China; 3 Tang Qiao Community Health Service Center of Shanghai, Shanghai 200010, PR China; 4 Department of Pathology and Laboratory Medicine, David Geffen School of Medicine, University of California at Los Angeles, CA 90095, USA

## Abstract

**Background:**

Osteoarthritis-induced arthralgia is a common cause of morbidity in both men and women worldwide. AR7 Joint Complex is a nutritional supplement containing various ingredients including sternum collagen II and methylsulfonylmethane. The product has been marketed in United States for over a decade, but clinical data measuring the effectiveness of this supplement in relieving arthralgia is lacking. The goal of this study was to determine the effect of AR7 Joint Complex on osteoarthritis.

**Methods:**

A total of 100 patients over the age of 50 who had osteoarthritis were recruited to the double-blind study and randomly assigned into either treatment or placebo control groups. The patients in the treatment group were given AR7 Joint Complex orally, 1 capsule daily for 12 weeks, while the patients in the control group were given a placebo for the same period of time. Prior to and at the end of the study, data including Quality of Life questionnaires (SF-36), visual analog scales (1 to 100 mm), and X-rays of affected joints were collected.

**Results:**

A total of 89 patients completed the study: 44 from the treatment group and 45 from the control group. No significant change in X-ray results was found in either group after the study. However, there was a significant decrease in patients complaining of arthralgia and tenderness (P < 0.01) in the treatment group and there was also a significant difference between the treatment and control groups at the end of the study. In addition, for Quality of Life data, the body pain index (BP) in the treatment group was significantly improved (P < 0.05) compared to the control group. No significant toxicity was noted in either group.

**Conclusion:**

AR7 Joint Complex appears to have short-term effects in relieving pain in patients with osteoarthritis. Whether such an effect is long-lasting remains to be seen.

## Introduction

Osteoarthritis (OA), or degenerative joint disease, is the most common form of arthritis and affects almost all joints, especially weight-bearing joints [1]. It affects both men and women of all races [2]. Its prevalence increases with age and is almost universal in individuals over the age of 75. In essence, it is a degenerative disease related to aging. Clinically, OA is characterized by progressive deterioration and loss of articular cartilage accompanied by proliferation of new bone and soft tissue in and around the involved joint [3]. Symptoms include joint pain, swelling, stiffness, and crepitus. The pain usually begins insidiously, in the form of a deep, aching, poorly localized pain occurring with the use of the involved joint and relieved by rest. Stiffness occurs in the morning and after periods of inactivity. Crepitus may occur with joint movement due to loss of cartilage. The most common clinical findings in physical examinations include the formation of Heberden's nodes (enlarged dorsomedial and lateral aspects of the distal interphalangeal joint) in the early stage and osteophytes, as well as severe deformity accompanied by joint sclerosis at a later stage [4].

The pathogenesis of OA is poorly understood, but inflammation appears to play an important role [5]. Breakdown products of cartilage stimulate the release of collagenase and other hydrolytic enzymes from cells in the synovium. The presence of immunoglobulin and complement in the superficial layer of cartilage suggest that immune complexes may induce an inflammatory response. Recently, cyclooxygenase-2 (COX-2) selective nonsteroidal anti-inflammatory drugs (NSAIDs), for example Celebrex, have been shown to be as effective as acetaminophen and nonselective NSAIDs in treating OA (for a recent review, see ref [6]). This finding highlights the importance of the inflammatory mechanism in the pathogenesis of this disease. There is no specific laboratory test to help establish the clinical diagnosis of OA. Radiographs of the involved joints are initially normal, but as the disease progresses, joint space narrowing, eburnation, and osteophytes are observed. The treatment includes the use of NSAIDs, intra-articular steroid injection, and orthopedic surgery [5].

AR7 Joint Complex is a nutritional supplement that has been available on the market for over a decade with no serious side effects reported. It contains sternum collagen, methylsulfonylmethane (MSM), cetyl myristoleate (CMO), lipase, turmeric, vitamin C, and bromelain. However, the benefits of this supplement in relieving symptoms of OA have not been tested in a clinical setting. The rationale of this project was to examine the short-term effects of AR7 in relieving symptoms, mainly joint pain and stiffness, in patients who suffer from OA. The findings might provide preliminary data to determine the benefits of AR7 as an alternative, non-surgical method for patients with OA.

## Methods

### Materials

AR7 in the form of softgels were provided by Robinson Pharma (Orange County, CA, USA). The product was manufactured following cGMP guidelines. The main ingredients of AR7 included sternum collagen, methylsulfonylmethane (MSM), cetyl myristoleate (CMO), lipase, vitamin C, and bromelain. The main ingredient of the placebo softgel was corn oil. In addition, there were gelatin, glycerin, purified water and titanium dioxide, and artificial food coloring. The placebo control pill was also manufactured by Robinson Pharma.

### Subjects

All subjects were identified and recruited from 2 community health centers in Shanghai, People's Republic of China: the Zha Pu community health service center and Tang Qiao community health service center. All patients were screened by American College of Rheumatology criteria [7]. The inclusion criteria included patients who were: over 50 years old at screening, either male or female, and displayed symptoms of degenerative joint diseases, including joint pain, stiffness, swelling, and difficulty walking and/or getting up/down stairs. All patients were free from the following diseases: cancer, gallstone, ulcers, and gout. None of them were taking bromelain, antibiotics (including amoxicillin and tetracycline), antiplatelet drugs, or warfarin. The study was reviewed and approved by the Internal Review Boards of the Zha Pu and Tang Qiao community health service centers, and consent was obtained from each of the study subjects.

According to the above stated inclusion/exclusion criteria, a total of 100 patients were recruited and randomly assigned into 2 groups: a treatment group and a control group, with 50 patients in each group. Among them, 11 patients withdrew from the study – 2 patients moved away, 2 decided to undergo physical therapy instead, 3 showed mild stomach discomfort after taking the pill within the first 2 weeks of the study, and the remaining 4 had difficulty making multiple hospital visits. As a result, 44 patients in the treatment group and 45 patients in the control group completed the study.

### Patient entry and randomization

Patients were enrolled in the study once the informed-consent form was signed. Each subject was blindly randomized into either the treatment (Group 1) or control group (Group 2). Simple randomization was conducted using random numbers from a computer-generated sequence. The randomization was conducted centrally in Dr. Shi's department at Shanghai Jiatong University. At the time of enrollment, each patient recruited for the study selected an envelope assigning him/her to one of the 2 groups. After randomization, each subject received a container marked with a colored label (e.g., red or blue) with 30 softgel capsules inside. Treatment blinding was maintained throughout the study period. Each subject took 1 color-coded capsule orally and daily with water, immediately after breakfast.

Physicians at each facility were responsible for performing clinical examinations, ordering and interoperating laboratory testing, and x-ray findings. They were also responsible for filling out the SF-36 form. They were trained by investigators at Shanghai Jiaotong University (Dr. Shi and Dr. Xie) prior to initiation of the study.

### Baseline assessment and laboratory testing

Each subject's date of birth, sex, race, medical history, current medications, and alcohol history were obtained by the investigators using a standard SF-36 questionnaire at the time of the initial screening prior to randomization. Physical examinations were performed by local physicians, focusing on joint conditions, and a standardized form was used to record findings. Laboratory tests were performed at the beginning and end of the study. Tests included: blood tests for CBC and Serum BUN/Creatinine; urine tests with clean-catch urine samples for dipstick analysis of hematuria and pH; and uric acid analysis (to rule out gout). X-rays of affected joints were also taken to document the degree of joint space narrowing, eburnation, and osteophytes.

### Study duration and patient follow-up

All study subjects were recruited within the first 2 weeks of the study. Each subject was then monitored for 3 months. The treatment period for each subject was exactly 3 months. Specific tests, as outlined above, were performed after the 2-week washout phase and again at the end of the study (3 months later). All subjects were asked to return to the clinic on a weekly basis for the first month and on a monthly basis for the remaining 3 months. At each visit, the investigators addressed concerns that the subject had, evaluated compliance and toxicity, and resupplied additional capsules. The capsules remaining in the returned bottle were counted and recorded. Records of other drugs taken at the same period were also taken.

### Quality of life measurement

The study used the standard "Health Survey" (SF-36) to measure quality of life of the studied subjects. The major components included physical function (or limitations of activity, PF), social function (or social activities, SF), physical health problems (PHP), emotional health problems (EHP), body pain (BP), vitality (VT), mental health (or energy and emotions, MH), and general health (GH). SF-36 surveys were conducted at the baseline and each follow-up visit by a physician was conducted in a blind fashion (without knowing if the subject was in the treatment or placebo group). All questionnaire data were coded according to severity from lowest to highest, and then summarized with one numerical score for each category where higher scores corresponded to better health status.

### Data analysis

The database was managed by the EpiData 3.0 system using SPSS 11.0 software for data analysis. For quantitative measurements, a Student's t-test was used to compare the results of the treatment and control groups while a paired t-test was used for comparing before-and-after data for each group. For qualitative data, chi-square analysis and the Wilcoxon Rank Sum test were used.

## Results

### Baseline data

The 89 subjects had an average age of 62.54 ± 9.05. They consisted of 21 males and 68 females. The control and treatment groups showed similar age compositions (mean age 63.27 ± 9.03 versus 61.82 ± 9.11, respectively) and gender distribution (Χ^2 ^= 2.85, P = 0.09).

The reported OA symptoms for each subject ranged from 1–39 years with an average of 9.10 ± 8.14 years. At the baseline, the 2 groups showed no significant differences in the length of time since onset of OA, history of taking steroid medication, and joint symptoms including joint pain, stiffness, tenderness, and limitation of activity (P > 0.05 for all, Table 1). Thus, the 2 groups were well-balanced. X-ray examinations also showed a similar degree of changes in joint narrowing, eburnation, and osteophytes in both groups (data not shown).

**Table 1:** Comparison of baseline percentages of positive findings in treatment and control groups

	Positive (%)			
			
Items	Treatment (n = 44)	Control (n = 45)	Χ^2 ^Value	P Value
Age	63.27	61.82		

Sex	M/F (15.9/84.1)	M/F (31.1/68.9)	2.852	0.091

X-ray findings*	100%	100%		

Taking medication	45.5	31.1	1.93	0.164

Joint pain	95.5	82.2	2.69	0.101

Joint stiffness	47.7	53.3	0.28	0.597

Joint tenderness	84.1	77.8	0.57	0.449

Joint activity limitation	61.4	46.7	1.93	0.164

*All patients recruited to the study showed similar degrees of x-ray findings including joint space narrowing, eburnation, and osteophytes.

### Results after intervention

After a 3-month study period, the percentage of patients reported to have joint pain, stiffness, and tenderness were significantly decreased in the treatment group versus the control group (Table 2). No serious or unexpected adverse events associated with the capsules were found. No significant difference was seen for limitation of activity between the 2 groups (Table 2) and X-ray data also did not show any significant changes between them (data not shown). Compared to the baseline (Table 1), however, the percentage of patients complaining of joint pain, stiffness, tenderness, and even limitation of activity were significantly decreased after the study in the treatment group, but not in the control group.

**Table 2:** Comparison of percentages of positive findings in treatment versus control groups after 12-week clinical study

	Positive(%)			
			
Items	Treatment (n = 44)	Control (n = 45)	Χ^2 ^Value	P Value
Joint pain	43.2*	71.1		

Joint stiffness	22.7*	42.2	3.85	0.050

Joint tenderness	45.5*	71.1	6.03	0.014

Joint activity limitation	27.3*	44.4	2.85	0.091

* P < 0.01 when comparing these variables before versus after the study in the treatment group.

When quality of life questionnaire data were analyzed item by item, we observed that there were no significant differences between the 2 groups at the baseline in any of the categories. After the study, there was a significantly improved score for body pain (BP) in the treatment group versus the control group (64.07 ± 14.22 versus 55.76 ± 18.00, respectively, P = 0.02, Table 3). Other categories including physical function (PF, or limitations of activity), physical health problems (PHP), vitality (VT), social function (SF, or social activities), emotional health problems (EHP), mental health (MH, or energy and emotions), and general health (GH) did not show a statistically significant difference between the two groups. In the treatment group, statistically significant improvements of scores were observed at the end of the study compared to the beginning for all categories except social function (SF) (P < 0.01 for all except SF by paired t-test, Table 3). However, such a change was also observed, although to a lesser a degree, in some categories (BHP, EHP, and GH) in the control group as well, suggesting some placebo effects might be present.

**Table 3:** Quality of Life for treatment group compared to control group before and after clinical study

	Before ($\bar{X}$ ± S)			After ($\bar{X}$ ± S)		
				
Items	Treatment (n = 44)	Control (n = 45)	P Value	Treatment (n = 44)	Control (n = 45)	P Value
PF	50.57 ± 25.00	52.44 ± 25.97	0.729	61.82 ± 26.31*	55.78 ± 25.69	0.276

PHP	40.10 ± 6.05	43.76 ± 6.52	0.690	54.55 ± 40.22*	58.89 ± 42#	0.636

BP	50.82 ± 14.36	51.64 ± 15.92	0.798	64.07 ± 14.22*	55.76 ± 18.00	0.020

VT	54.55 ± 15.58	58.78 ± 14.89	0.194	58.41 ± 14.21*	61.89 ± 15.79	0.278

SF	73.99 ± 16.24	69.63 ± 17.63	0.229	74.24 ± 13.51	77.28 ± 18.49	0.379

EHP	16.29 ± 19.85	24.81 ± 23.20	0.066	53.03 ± 47.31*	52.60 ± 45.22*	0.965

MH	60.82 ± 13.46	66.40 ± 16.52	0.085	65.64 ± 13.18*	67.91 ± 16.35	0.472

GH	49.54 ± 14.45	52.43 ± 15.94	0.373	61.68 ± 19.68*	61.44 ± 19.85*	0.955

*: P < 0.01;

#: P < 0.05.

PF: physical function (or limitations of activity); PHP, physical health problems; BP, body pain; VT, vitality; SF, social function (or social activities); EHP, emotional health problems, MH, mental health (or energy and emotions); and GH, general health.

## Discussion

OA is a common disease affecting middle-aged to elderly people [3]. Though the mortality rate of OA may be low, the morbidity and effect on quality of life can be quite substantial. Research has shown that people with OA not only have reduced body function and social function, but also low moods, pain, fatigue, and reduced quality of life [8]. People with joint disease have a tendency to be depressed when compared to healthy individuals [9]. Currently, the main treatments for OA are nonsteroidal anti-inflammatory drugs (NSAIDs). But these treatments cannot inhibit OA degeneration. In addition, long-term use of these drugs has shown many side effects.

AR7 Joint Complex is a dietary supplement containing sternum collagen type II, methylsulfonylmethane (MSM), cetyl myristoleate (CMO), vitamin C, bromelain, turmeric, and lipase. Collagen type II is the major ingredient in this nutritional supplement. As a major component of joint cartilage, it is important in maintaining joint function. A study has shown that collagen type II may suppress the local immune response, which may delay cartilage degeneration and inhibit chronic inflammation [10]. Interestingly, Whitacre reported that OA patients misdiagnosed with rheumatoid arthritis showed significantly improved joint symptoms after taking collagen type II [11]. Yet another study reported that collagen type II might inhibit the cartilage matrix reductase, suggesting that it may reduce the joint degeneration seen in OA patients [12].

Other ingredients contained in AR7 Joint Complex such as MSM, CMO, bromelain, turmeric, lipase, and vitamin C are widely considered to have some potential beneficial effects on joint disease, but the evidence is less clear. For example, MSM provides a rich source of sulfur, which is a required structural mineral found in connective tissue including mucopolysaccharides and fibrous cartilage that maintain elasticity and flexibility [13]. CMO, on the other hand, is a fatty acid ester that serves as a surfactant to lubricate the involved joints [14]. Bromelain is derived from pineapple and it is believed to be a smooth muscle relaxant, helping to relieve cramping, alleviate joint discomfort and swelling, and increase joint mobility [14]. Turmeric (*Curcuma longa*) is a perennial herb of the ginger family, which has been used in Ayurvedic medicine to decrease redness and swelling [15]. However, few scientifically sound reports are available to substantiate the claims of these components.

The results of the current study show that after taking AR7 Joint Complex the treatment group had a significantly decreased percentage of patients complaining of joint pain and tenderness compared to the control group (Table 2). Significant improvement was also observed when comparing pre- and post-study data in the treatment group for joint pain, stiffness, tenderness, and limitation of activity. No significant improvement was noted in the control group. A similar trend was also observed when quality of life data were analyzed item by item (Table 3). Together, these findings suggest that there is evidence that AR7 Joint Complex may provide anti-arthralgia effects in OA patients. However, these effects were observed when comparing the data from the baseline to the end of the three-month study. At this time, we do not know how long it takes to achieve the effects, nor do we know how long they will last either with or without continued application of AR7 Joint Complex. But it is clear from this short-term study that no significant changes of X-ray findings are seen with the short-term treatment. This may be due to the fact that many of the patients studied have long-standing history of OA (average 9 years) and such a short-term treatment would be unlikely to have a substantial effect on reversing the structural damage. Alternatively, AR7 Joint Complex may only provide anti-inflammatory effects that have little to do with degenerative joint changes.

There were some significant improvements in several variables for quality of life data in the control group (PHP, EHP, and GH) before and after the study, although the magnitude of improvement was substantially smaller compared to the treatment group for each variable (Table 3). This finding suggests the presence of a placebo effect. However, since there was no significant change in the percentage of patients reporting joint symptoms before and after the study in the control group overall, such a placebo effect should be rather limited.

The exact pain-relieving mechanisms of AR7 Joint Complex in the OA patients remain to be determined. AR7 Joint Complex may function in the following ways: 1) it may help correct or balance enzyme activities, thereby inhibiting the activities of cartilage matrix degeneration; 2) it may enhance joint flexibility by lubricating joints and relaxing surrounding muscles; 3) it may have an anti-inflammatory effect in the joint.

While some short-term benefits of AR7 in relieving symptoms of OA have been observed in this placebo-controlled randomized study, it should be noted that the sample size was relatively small and the duration was short. Therefore, caution should be taken into consideration when interpreting the data, and additional studies with larger sample sizes and longer-term treatment may be necessary to test the effectiveness of AR7 on OA patients.

## Conclusion

In summary, this short-term placebo-controlled study showed that the nutritional supplement AR7 Joint Complex can help alleviate pain associated with OA. However, the study is limited by a small sample size and short-term treatment. Long-term effects and the actual mechanisms remain to be studied.

## Authors' contributions

Each author has contributed substantially to the study, including study design, data collection, data analysis, and manuscript preparation.
